# Cohabitation Histories of U.S. Older Adults

**DOI:** 10.1093/geroni/igaf122.840

**Published:** 2025-12-31

**Authors:** Susan Brown

**Affiliations:** Bowling Green State University, Bowling Green, Ohio, United States

## Abstract

The rapid acceleration in unmarried cohabitation during the second half of the 20th century reshaped the family life course as cohabitation shifted from the margins to being the modal path to marriage. Today’s older adults came of age during an era marked by low levels of cohabitation. But as they aged into midlife and beyond, cohabitation grew in popularity, diffusing across the population. As remarriage after divorce plummeted, a corresponding increase in cohabitation largely offset this decline. Cohabitation is now a common living arrangement among all age groups, including older adults. This diffusion process raises important questions about the levels and patterns of cohabitation experienced by today’s older adults over their life courses. Drawing on three waves of data from the National Social Life, Health, and Aging Project (NSHAP), a national sample of adults aged 57-85 begun in 2005-06 and refreshed in 2015-16, we use demographic techniques to describe the cohabitation histories of older adults, tracking variation by sex and across birth cohorts (born 1920-1965). We perform a series of life table estimates of having ever cohabited by various ages as well as having ever experienced premarital cohabitation, postmarital cohabitation, and serial cohabitation. We also assess the disposition of cohabiting unions to determine whether they eventuated in marriage or separation. Our study extends the growing literature on the marital biographies of older adults by integrating cohabitation histories, providing a more comprehensive portrait of the union experiences of U.S. older adults.

